# Epidemiological characteristics of hand, foot, and mouth disease in Yunnan Province, China, 2008–2019

**DOI:** 10.1186/s12879-021-06462-4

**Published:** 2021-08-04

**Authors:** Li Jiang, Hongchao Jiang, Xin Tian, Xueshan Xia, Tian Huang

**Affiliations:** 1 Kunming Children’s Hospital, Kunming, Yunnan People’s Republic of China; 2 Kunming University of Science and Technology, Kunming, Yunnan People’s Republic of China; 3 Yunnan Provincial Center for Disease Control and Prevention, 158 Dongsi Street, Kunming, Yunnan 650022 People’s Republic of China

**Keywords:** Hand, Foot, And mouth disease, Enterovirus 71 vaccine, Epidemiology

## Abstract

**Background:**

Since 2016, enterovirus 71 (EV71) vaccines have been approved for market entry, and little is known about how the epidemiology of hand, foot, and mouth disease (HFMD) has been affected by the introduction of the vaccines in Yunnan Province. The study describes the epidemiological characteristics of HFMD before and after the introduction of EV71 vaccination in Yunnan Province.

**Methods:**

Surveillance data collected between 2008 and 2019 were analyzed to produce epidemiological distribution on cases, etiologic composition, and EV71 vaccination coverage, as well as to compare these characteristics before and after EV71 vaccination.

**Results:**

A total of 1,653,533 children received EV71 vaccines from 2016 through 2019 in Yunnan. The annual EV71 vaccination coverage rate ranged from 5.53 to 15.01% among children ≤5 years old. After the introduction of EV71 vaccines, the overall incidence of HFMD increased and reached over 200 cases per 100,000 population-years in 2018 and 2019. However, the case severity and case fatality rate decreased and remained lower than 1 and 0.005% after 2016, respectively. EV71-associated mild, severe and fatal cases sharply decreased. The predominant viral serotype changed to non-EV71/non-CV-A16 enteroviruses which were detected across the whole province.

**Conclusions:**

Non-EV71/non-CV-A16 enteroviruses became the predominant strain and led to a higher incidence in Yunnan. Expanding EV71 vaccination and strengthening laboratory-based surveillance could further decrease the burden of severe HFMD and detect and monitor emerging enteroviruses.

**Supplementary Information:**

The online version contains supplementary material available at 10.1186/s12879-021-06462-4.

## Introduction

Hand, foot, and mouth disease (HFMD) is a viral illness mostly seen among children under 5 years old in the West Pacific, that causes mild symptoms including fever, erythra, vesiculation, and inappetence. However, some symptoms are severe and involve neurological complications and can even lead to death [1]. HFMD has been categorized as a class C notifiable disease in China since May 2, 2008. By the end of 2015, over 13 million HFMD cases were reported, including 123,261 severe cases and 3322 deaths in mainland China. Laboratory surveillance results showed that the causes of HFMD are enterovirus 71 (EV71) and coxsackievirus A16 (CV-A16) in most cases. Moreover, EV71 is the most frequently identified serotype among both severe and fatal cases [2].

Currently there is no specific antiviral treatment for HFMD. Three inactivated monovalent EV71 vaccines from Beijing Vigoo Biological Co., Ltd. (Vigoo), Sinovac Biotech Co., Ltd. (Sinovac), and the Institute of Medical Biology, Chinese Academy of Medical Sciences (Kunming Institute) were recently licensed in China, which showed high efficacy (90.0–97.4%) against EV71-associated HFMD but no cross-protection against HFMD caused by CV-A16 or other serotypes in children [3–5].

Since 2016, EV71 vaccines have been introduced in Yunnan Province, China [6]. The vaccine is voluntary and self-paid. The cost of the vaccine is around 200 China Yuan (CNY) per dose. It was recommended that children aged of 6 months to 5 years old receive the first dose, as well as a second dose on day 28. However, the impact of this new vaccine on the epidemiology of HFMD is currently unknown after the introduction of EV71 vaccines in the province. Thus, we accessed the surveillance data of HFMD reported from 2008 through 2019 in Yunnan in an attempt to describe the epidemiological features of HFMD patients following the introduction of EV71 vaccines.

## Method

### Study area

Yunnan is a province of China located in the most southwest of the country. It spans approximately 394,000 km^2^ and has a population of 48 million (2019) [7]. We divided the province into 4 regions, including central, northeastern, northwestern, and southern areas, according to proximity, culture, and socioeconomic status. More than half of the population lives in the central and northeastern parts of the province, which accounts for 30% of the total area of the province.

### Data sources

Data on HFMD cases from 1 January 2008 to 31 December 2019 were obtained from the National Surveillance of Notifiable Infectious Disease Programme (NSNIDP), which was constructed by the China Centers for Disease Control and Prevention (China CDC). HFMD was required as a notifiable infectious disease to be reported to the NSNIDP since May 2, 2008. Basic demographic and diagnostic information and etiological results of HFMD cases and population by age group were collected via the NSNIDP. The vaccinated population in Yunnan was extracted from the Immunization Planning Information Management System (IPIMS) at the Yunnan CDC. When registration in the NSNIDP and IPIMS, written informed consent was obtained from each adult patient and legally authorized representatives of patient who was under 18 year of age. Data obtained from the NSNIDP and the IPIMS were anonymized and did not include any personal data.

According to HFMD diagnostic criteria, cases were categorized as severe when they presented more than one of the following complications: encephalitis, aseptic meningitis, or acute flaccid paralysis, pulmonary edema, pulmonary hemorrhage, or myocarditis, otherwise as mild.

Virological surveillance was conducted by local CDC. Specimens were collected from cases and sent to laboratories for coxsackie virus A 16 (CV-A16) and enterovirus 71 (EV71) and other enterovirus nucleic acid testing by polymerase chain reaction or virus isolation. All methods were performed in accordance with the relevant guidelines and regulations that have been described in a previous study [8].

### Statistical analysis

All data in this study were transferred into the R program (version 3.2.1) [9] for data exploration and analysis. The incidence rate of HFMD was calculated by dividing the number of all reported HFMD cases by the total person-year in the corresponding time. The case severity rate and case fatality rate were identified as the proportion of severe cases and fatal cases among all reported cases, respectively. The seasonal pattern was described by year and month. The geographic distributions were marked by the four regions, including the central, northeastern, northwestern, and southern areas of Yunnan. The demographic characteristics, including gender and age, were presented as percentages and medians by pie and box graphs, respectively. Annual virological surveillance results were presented in percent bar graphs to explore the viral serotype epidemic cycle.

Furthermore, as individual vaccination data were lacking, the annual vaccination coverage rate (2016–2019) was estimated as the proportion of EV71 vaccinated people among children ≤5 years old in Yunnan. The EV71 vaccine was launched in Yunnan Province in 2016. Thus, 2017–2019 as the implementation period of the EV71 vaccination program to reflect the changes in HFMD epidemiology post-EV71 vaccine introduction. 2008–2015, as the baseline years before EV71 vaccination, was identified as pre-EV71 vaccine introduction. Epidemiological information on incidence, patient demographics, clinical classifications and viral serotypes was compared between before and after EV71 vaccine introduction. The significance of differences was initially assessed with the Χ^2^ test and rank sum test.

## Results

### Incidence, severity, and fatality rates of HFMD

**Fig. 1:**
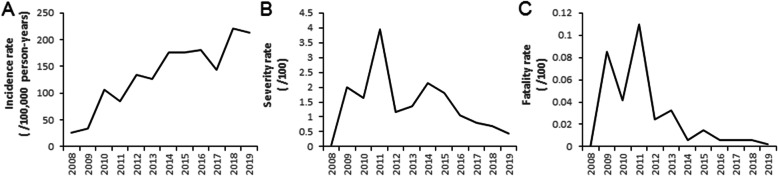
Annual incidence rates (per 100,000 population), severity rates (per 100 cases), and fatality rates (per 100 cases) of reported hand, foot and mouth disease patients in Yunnan Province 2008–2019

From 2008 through 2019, a total of 763,863 HFMD cases were reported in the NNIDRS. Of these cases, 9928 cases were diagnosed as severe and included 144 deaths. The incidence of reported HFMD cases showed an upward trend since the initiation of surveillance in 2008 and reached a peak of over 200 cases per 100,000 population-years in 2018 (Fig. 1a). Both the case severity rate (Fig. 1b) and case fatality rate (Fig. 1c) presented large fluctuations during 2008–2015. However, the two rates continued to show downward trends and remained at lower levels after 2016. Compared to the pre-EV71 vaccine introduction period (2008–2015), there were significant changes in the incidence, severity, and fatality rates in the post-EV71 vaccine introduction period (2017–2019) (S Table 1).

### Seasonal pattern

**Fig. 2:**
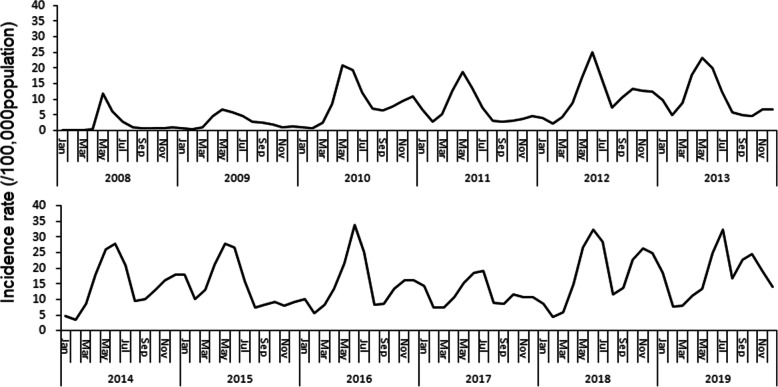
Incidence rates (per 100,000 population) of hand, foot and mouth disease in Yunnan Province by month 2008–2019

HFMD was prevalent throughout the year in Yunnan, there was a single peak in May in 2008 and 2009. Since 2010, annual epidemic waves with a major peak in the early summer (May) followed by a smaller peak in autumn (November) were observed. However, the scales of the autumn peaks in 2013, 2015, 2017, and 2019 were smaller than those in the corresponding previous year (Fig. 2).

### Geographic distribution

**Fig. 3:**
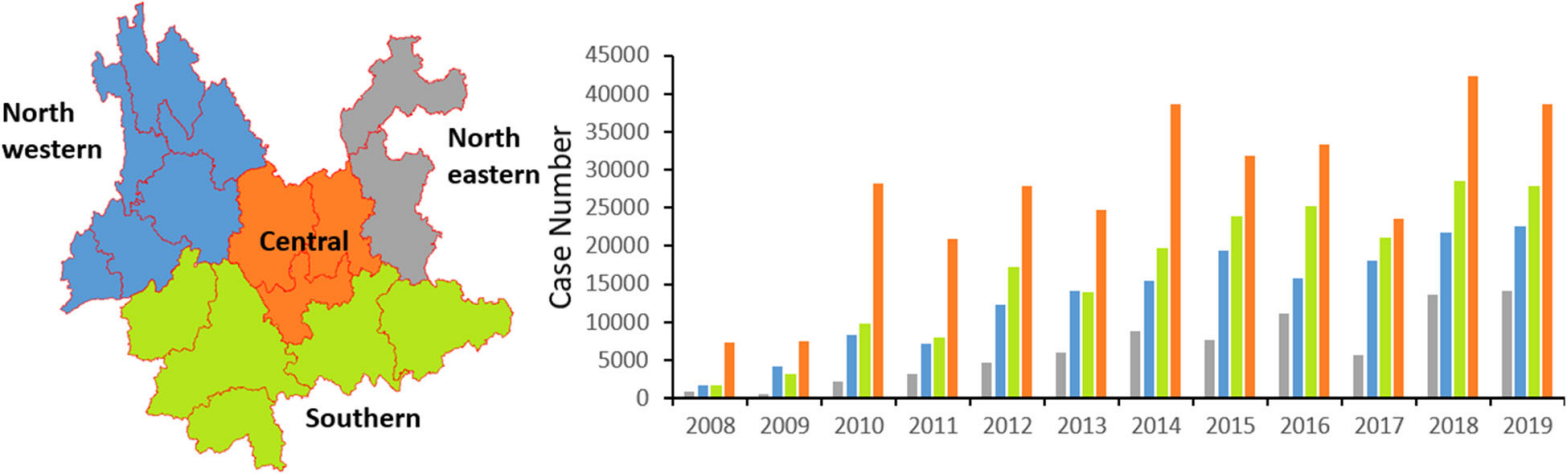
Reported cases of hand, foot and mouth disease in Yunnan Province by area 2008–2019

Of all cases reported, 324,822 (42.52%), 200,107 (26.20%), 160,773 (21.05%), and 78,161 (10.23%) cases were diagnosed from the central, southern, northwestern, and northeastern regions of the province during the surveillance period, respectively. Similar patterns of case geographic distribution were presented in each year (Fig. 3).

### Characteristics of age and gender

**Fig. 4:**
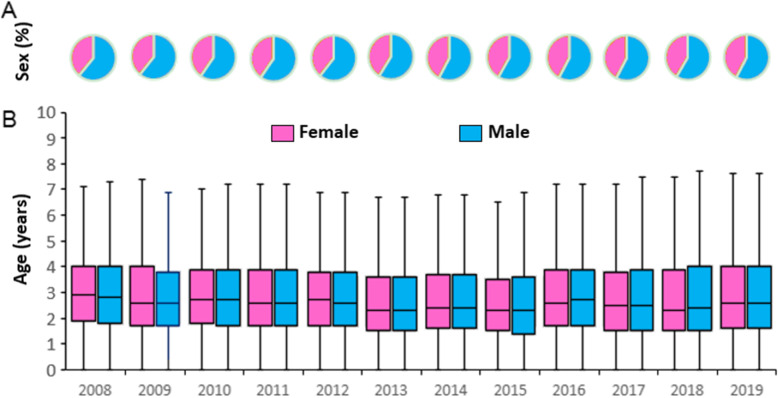
Sex composition and age distribution of reported hand, foot and mouth disease patients in Yunnan Province by year 2008–2019

There were 446,942 male and 316,921 female cases, as indicated by a male-to-female ratio of 1.41:1 among the reported cases. The proportions of case stratified by sex in each year are depicted by a pie graph (Fig. 4a). Males accounting for more than half of the cases were observed in each surveillance year. The distributions of age are shown by year and sex using box plots (Fig. 4b). The median age ranged from 2.3 to 2.9 years during the surveillance years.

### Etiologic composition

**Fig. 5:**
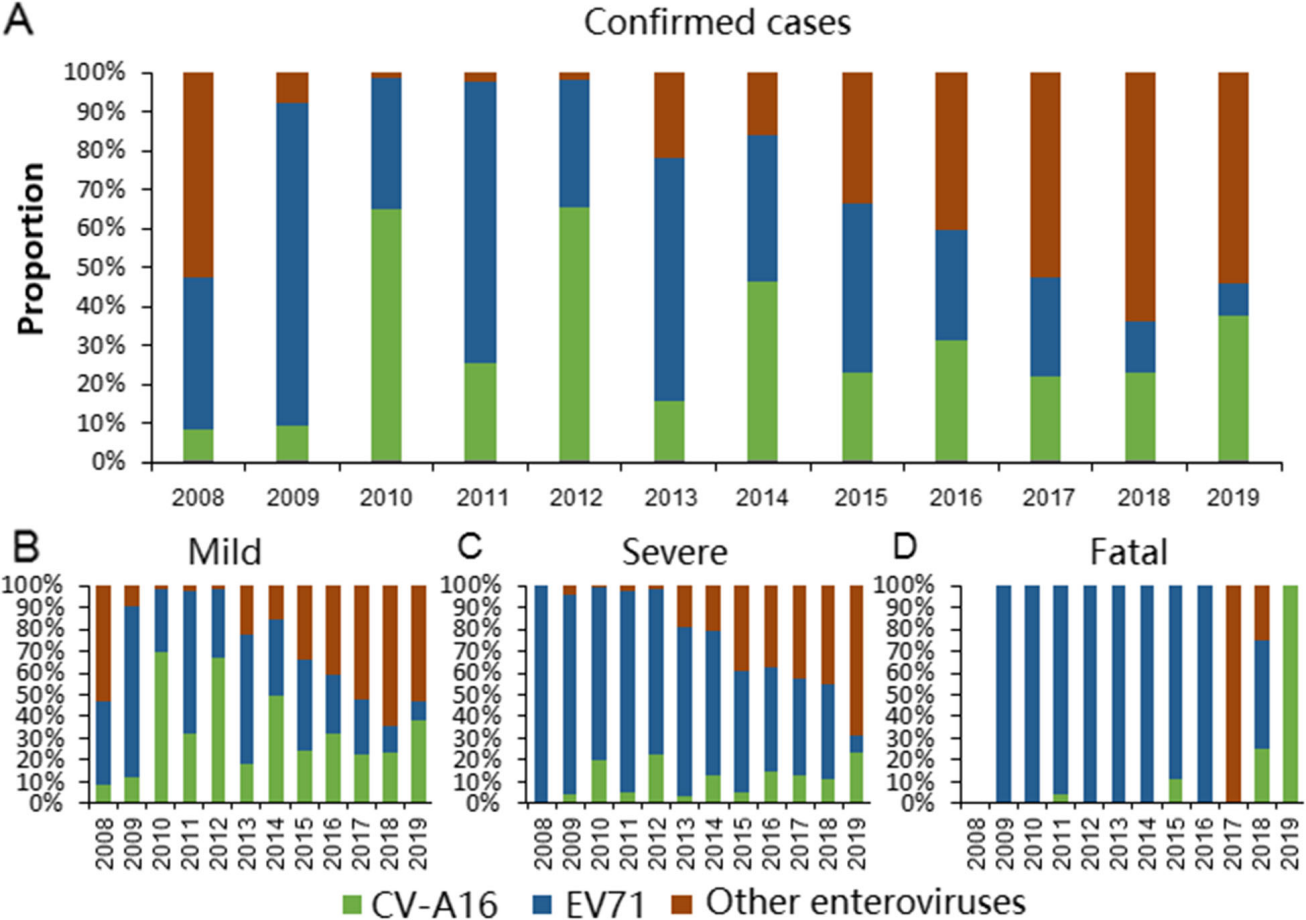
Proportions of enterovirus serotypes among laboratory-confirmed, mild, severe, and fatal cases of hand, foot, and mouth disease in Yunnan Province by year 2008–2019

Laboratory tests confirmed 65,319 cases during 2008–2019, including 61,120 mild cases and 4199 severe cases, of which 75 were fatalities. Among the laboratory-confirmed cases, 20,475 (31.35%), 22,586 (34.58%), and 22,258 (34.07%) cases were associated with EV71, CV-A16 and other enteroviruses, respectively (Fig. 5a). EV71 was the predominant serotype for mild cases in 2009, 2011, 2013, and 2015. CV-A16 was the predominant serotype among mild cases in 2010, 2012 and 2014. However, since 2016, other enteroviruses occupied the largest proportion among mild cases annually (Fig. 5b). Most severe cases were caused by EV71. However, other enteroviruses have accounted for an increasing proportion of severe cases since 2013. In 2019, more than half of severe cases were associated with other enteroviruses (Fig. 5c). EV71 was the major pathogen among fatal cases. However, other enteroviruses and CV-A16 were the predominant serotypes among fatal cases in 2017 and 2019, respectively (Fig. 5d).

### EV71 vaccination rate

A total of 1,653,533 people received the EV71 vaccine from 2016 through 2019 in Yunnan. The annual vaccination rate ranged from 5.53 to 15.01% among children ≤5 years old. And it showed an upward trend during the study period (Table 1).

**Table 1:** The EV71 vaccination coverage rate among children (≤5 years old) in Yunnan Province, 2016–2019

Year	Number of people vaccinated	Population	Annual coverage rate (%)
2016	192,700	3,482,682	5.53
2017	455,630	3,397,251	13.41
2018	471,911	3,221,882	14.65
2019	533,292	3,552,676	15.01

### Changes in HFMD epidemiology before and after EV71 vaccine introduction

Compared to the pre-EV71 vaccine introduction period (2008–2015), there was significant decrease in male cases in the post-EV71 vaccine introduction period (2017–2019). The median age (2.5 years, IQR: 1.6–4.0) of cases during the post-vaccine period was significantly higher than that (2.3 months, IQR: 1.5, 3.6) of cases during the pre-vaccine period. The number of cases decreased evidently among the age groups of under 3 years. All four regions of the province had an increase in the number of cases. More evident increasing trends were observed in the southern, northeastern, and northwestern areas. However, severe and fatal cases showed a significant decrease during the post-vaccine period. The proportion of HFMD cases caused by non-EV71/non-CV-A16 enteroviruses increased significantly. EV71-associated cases sharply decreased during the post-vaccine period (Table 2 and S Table 2).

**Table 2:** Comparison of epidemiological characteristics between before (2008–2015) and after (2017–2019) introduction of EV71 vaccines in Yunnan Province

Items	Pre-vaccine introduction (2008–2015) *n* = 400,704	Post-vaccine introduction (2017–2019) *n* = 277,731	X^2^ test *P* value
Sex			< 0.01
Female	164,082 (40.95)	116,755 (42.04)	
Male	236,622 (59.05)	160,976 (57.96)	
Age			< 0.01
0–0.5	4647 (1.16)	2909 (1.05)	
0.6–2.9	229,931 (57.38)	157,810 (56.82)	
3–6.9	149,927 (37.42)	104,597 (37.66)	
7–14.9	15,049 (3.76)	11,223 (4.04)	
≥ 15	1150 (0.29)	1192 (0.43)	
Median (IQR)	2.5 (1.6,3.7)	2.5 (1.6, 4.0)	< 0.01^a^
Area			< 0.01
Central	187,087 (46.70)	104,454 (37.61)	
Northeastern	33,648 (8.40)	33,311 (11.99)	
Northwestern	82,590 (20.60)	62,432 (22.48)	
Southern	97,379 (24.30)	77,534 (27.92)	
Case classification			< 0.01
Mild	393,261 (98.14)	275,995 (99.37)	
Severe	7316 (1.83)	1724 (0.62)	
Death	127 (0.03)	12 (0.01)	
Pathogen			< 0.01
EV71	14,208 (44.1)	3239 (14.41)	
CV-A16	12,987 (40.3)	6307 (28.06)	
Others	5052 (15.7)	12,929 (57.53)	

*IQR* interquartile range

^a^Rank sum test

## Discussion

The annual vaccination coverage rate ranged from 5.53 to 15.01% among children ≤5 years old in Yunnan during the last 4 years. However, as estimated from the Chengdu study, to build herd immunity for ending EV71-related HFMD transmission, the vaccination coverage levels should reach 94.0% among infants [10]. Parental knowledge of the vaccine, adverse effects and cost were the most important factors that influenced willingness for EV71 vaccination uptake [11–13]. Yunnan is one of the undeveloped provinces in both the economy and culture in China [8]. To expand EV71 vaccination, more efforts on these influencing factors should be made by the local government.

The EV71 virus is divided into four genotypes (A, B, C and D) and is further divided into 12 subgenotypes. The current inactivated vaccine was derived from the C4 viral strain. The cross-protection effect of vaccines against various virus strains has not been clearly identified [14]. In a previous study, all of the EV71 isolates from Yunnan belonged to the C4 genotype [15, 16]. The vaccine was highly matched with the predominant viral strains in the province. However, Yunnan Province is a border province of China and neighbors Southeast Asian countries, all of which are HFMD-endemic areas. Annual large cross-border population movement creates great challenges for preventing the transmission of infectious diseases in the province. HFMD was the most common gastrointestinal infection among imported cases in the border area of Yunnan [17]. EV71 genotypes C4, C2 and B5 have been demonstrated to cocirculate in Thailand and Vietnam [18, 19]. Hence, monitoring changes in EV71 epidemic strains in the border area of the province is necessary to prevent the cross-border spread of these novel viral strains.

The present study showed ongoing epidemiological trends in HFMD since the disease was involved in NSNIDP in 2008. Following the introduction of EV71 vaccines in the province, case severity and fatality rates have been reduced, while the incidence rate has evidently increased. Similar findings have been reported in Chengdu and Guangzhou cities [10, 20]. Virological surveillance indicated that non-EV71/non-CV-A16 enteroviruses contributed to the increase in incidence rate. However, the decrease in case severity and fatality was primarily attributed to the EV71 virus. The evidence strongly supported the beneficial impact of EV71 vaccination on the control of severe HFMD. However, the proportion of severe and fatal cases caused by other non-EV71 viruses had a significant increasing trend in Yunnan during the period of post-EV71 vaccine introduction. Thus, non-EV71 virus-associated HFMD should be highlighted to understand clinical manifestations to avoid disease progression.

Since 2013, the proportion of non-EV71/non-CV-A16 enteroviruses rose markedly, and it was greater than 50% after 2017 in Yunnan. However, because most non-EV71/non-CV-A16 enteroviruses were not identified for specific serotypes, the current predominant viral strain was not well understood in the province. The whole etiological spectrum following EV71 vaccination in Xiangyang revealed that CV-A6 was the predominant serotype, followed by CV-A16, CV-A10, CV-A5, CV-A2 and EV71 [21]. However, since 2012 or even earlier, CV-A6 and CV-A10 have been the predominant enterovirus serotypes, overtaking EV71 and CV-A16 causing mild, severe and fatal cases of HFMD in some areas in China [22–24]. Moreover, an active virological investigation prior to the introduction of the EV71 vaccine in Yunnan showed CV-A6, CV-A10 and echovirus (E)-9 were the three most frequent serotypes among non-EV71/non-CV-A16 enteroviruses. CV-B5, CV-A9, E-30, E-18, CV-A4, C-B3, CV-A2 CV-A8, CV-A14, E-14, E-11, and CV-B4 were also detected [15]. These findings may indicate that current circulating non-EV71/non-CV-A16 serotypes can occur independently and may not be a result of EV71 vaccine selective pressure. Thus, long-term and expanding active etiological surveillance of HFMD is needed to understand the effect of EV71 vaccination on HFMD epidemiology and to reveal the existence of emerging viruses.

The seasonal pattern of HFMD presented yearly double peaks in Yunnan from 2017 to 2019. The two peak patterns were not changed by the introduction of the EN71 vaccine. Climatological factors including temperature and humidity, can induce EV71 and CV-A16 to cause differing epidemic scales [25]. Lager scales of the autumn peaks were detected in 2018 and 2019 in our study. Whether the changes in seasonal peaks are attributed to the changing serotype distribution combined with climate interruption needs to be investigated in further studies.

HFMD infections showed an increasing trend among all age groups after the introduction of EV71 vaccines in Yunnan. This finding was similar to the observation in Chengdu, which may be attributed to the significant increase in circulating non-EV71 enteroviruses [10]. The median age of HFMD infection was slightly delayed in our study. The Guangzhou study showed significant protection from the EV71 vaccine for HFMD among children 3 years of age [20]. Whether the delayed infection age was caused by the impact of EV71 vaccination or circulating non-EV71 enteroviruses, investigation on age-specific incidence by viral serotypes and vaccination rate should be carried out in further studies.

The main limitation of this study was that the demographics of children who were vaccinated were not all recorded in the CDC vaccination surveillance system, because the EV71 vaccine is voluntary and self-paid in China. Thus, we could not estimate the age-specific vaccination coverage rate.

## Conclusion

In conclusion, ongoing epidemiological changes in HFMD following the introduction of the EV71 vaccine were observed in Yunnan. Non-EV71/non-CV-A16 enteroviruses became the predominant strain and brought larger scale incidence. Expanding EV71 vaccination and strengthening laboratory-based surveillance could further decrease the burden of severe HFMD and detect and monitor emerging enteroviruses.

## Supplementary Information

**Additional file 1: S Table 1.** Comparison of the average incidence, severity, and fatality rate between before (2008–2015) and after (2017–2019) introduction of EV71 vaccines in Yunnan Province.

**Additional file 2: S Table 2.** Comparison of the case severity and etiology in before (2008–2015) and after (2017–2019) introduction of EV71 vaccines in Yunnan Province.

## Data Availability

The datasets used and analyzed during the current study are available from the corresponding author on reasonable request.

## Abbreviations

CV-A16: Coxsackievirus A16
E-9: Echo virus-9
EV71: Enterovirus 71
HFMD: Hand, foot, and mouth disease
NSNIDP: National Surveillance of Notifiable Infectious Disease Programme
IPIMS: Immunization Planning Information Management System
