# Supplementary material for: Epidemiological characteristics of hand, foot, and mouth disease in Yunnan Province, China, 2008–2019
Source: BMC Infect Dis. 2021 Aug 4;21:751. doi: 10.1186/s12879-021-06462-4 (PMC8336324; doi:10.1186/s12879-021-06462-4)
Supplement: Supplementary file 1 — Additional file 1: S Table 1. Comparison of the average incidence, severity, and fatality rate between before (2008–2015) and after (2017–2019) introduction of EV71 vaccines in Yunnan Province. [file 12879_2021_6462_MOESM1_ESM.docx]

S Table 1

Comparison of the average incidence, severity, and fatality rate between before (2008-2015) and after (2017-2019) introduction of EV71 vaccines in Yunnan Province

| Items | Pre-vaccine introduction  (2008-2015) | Post-vaccine introduction  (2017-2019) | X^2^ test  P value |
| --- | --- | --- | --- |
| Incidence (/100,000) | 110.9616 | 191.6711 | <0.01 |
| Severity (%) | 1.8575 | 0.6251 | <0.01 |
| Fatality (%) | 0.0317 | 0.0043 | <0.01 |
