# Supplementary material for: Epidemiological characteristics of hand, foot, and mouth disease in Yunnan Province, China, 2008–2019
Source: BMC Infect Dis. 2021 Aug 4;21:751. doi: 10.1186/s12879-021-06462-4 (PMC8336324; doi:10.1186/s12879-021-06462-4)
Supplement: Supplementary file 2 — Additional file 2: S Table 2. Comparison of the case severity and etiology in before (2008–2015) and after (2017–2019) introduction of EV71 vaccines in Yunnan Province. [file 12879_2021_6462_MOESM2_ESM.docx]

S Table 2

Comparison of the case severity and etiology in before (2008-2015) and after (2017-2019) introduction of EV71 vaccines in Yunnan Province

| Time period | virus | Mild (%) | Severe (%) | Fatal (%) | [Fisher's exact test](http://www.baidu.com/link?url=jU0ek-ZK1FXVitCjz0hCJSkeLwBuc3ICWIT8zS7I6Y6glFzmfEQf_fpybCaFA3Y03SyRkHkv4TfTAwilAg8aE2uxfFRCYTgRgM19IN6uGPy)  P value |
| --- | --- | --- | --- | --- | --- |
| 2008-2015 | EV71 | 11876  (40.71) | 2267  (75.34) | 65  (97.01) | <0.01 |
|  | CV-A16 | 12707  (43.56) | 278  (9.24) | 2  (2.99) |  |
|  | Other enteroviruses | 4588  (15.73) | 464  (15.42) | 0  (0) |  |
| 2017-2019 | EV71 | 3083  (14.16) | 154  (22.00) | 2  (33.33) | <0.01 |
|  | CV-A16 | 6173  (28.36) | 132  (18.86) | 2  (33.33) |  |
|  | Other enteroviruses | 12513  (57.48) | 414  (59.14) | 2  (33.33) |  |
